# The money laundering market: An economic analysis

**DOI:** 10.1073/pnas.2512080123

**Published:** 2026-08-10

**Authors:** Joras Ferwerda

**Affiliations:** ^a^ School of Economics, Utrecht University, Utrecht 3508 TC, The Netherlands

**Keywords:** money laundering, illegal markets, market analysis

## Abstract

Criminals launder their proceeds from crime, either themselves or by paying a professional, to disguise the link between the criminal, the crime, and the proceeds. This allows the criminal to spend the proceeds more freely and lowers the chance of receiving unwanted attention from the authorities. This paper investigates the structure and dynamics of the understudied market for money laundering services. The analysis reveals that this illegal market is demand-driven and fragmented, with different forms of laundering, each requiring different steps with varying levels of risk. High search costs, specialized money launderers, and uncertainties lead to local monopolies. Demand tends to be relatively inelastic because criminals eventually need laundering services to spend their proceeds. Local monopolies, inelastic demand, and poor information flows lead to relatively high prices for laundering. Trust issues and two-sided asymmetric information lead to a market with relationships that tend to last longer than generally expected in illegal markets.

Spending criminal proceeds on anything of substantial value risks drawing unwanted attention from the authorities. Money laundering gives such criminal proceeds an apparent legal source or at least makes it difficult to understand the link between the criminal, the crime, and the money.^*^ Once the money is successfully laundered, it can be spent more freely. This makes money laundering a valuable service for criminals with significant proceeds who cannot or do not want to do the laundering themselves.

Criminals need to interact with the legitimate economy when laundering money; they have to come up for air, so to speak. This can provide an opportunity to catch criminals who have so far been able to stay undetected. The Medellín Cartel in the 1980s was having trouble finding money laundering providers. The cartel ended up being susceptible to laundering services offered by undercover US police agents, which resulted in a great success story for law enforcement (Operation Polar Cap, see ref. 1). Criminalizing money laundering gives law enforcement agencies this extra angle of attack to mitigate the negative effects of crime.

This paper conducts an economic analysis of the illegal market for laundering services by examining market features such as market size, relevant parties, demand and supply, prices, quality, and competition.

This paper argues that the money laundering market is fragmented. The many different laundering methods require different laundering steps, with varying levels of legal and procedural risks. There tend to be local monopolies in the laundering market, owing to high search costs, uncertainties, and specialization by launderers who focus their laundering services on proceeds from specific crimes. Criminals with large earnings, at some point, need laundering services to spend their ill-gotten gains, which leads to relatively inelastic demand. The local monopolies, inelastic demand, and poor information flows can lead to relatively high prices for money laundering. Owing to trust issues and two-sided asymmetric information (the criminal and the launderer know something the other does not know), we can expect money laundering relationships to be less ephemeral than generally expected in other illegal markets (2).

Successfully fighting money laundering might not always directly affect the volume of laundering, since criminals still need to launder their money somehow. However, effectively fighting money laundering based on a good understanding of the market can increase the price of laundering and therefore increase the cost for criminals. This would make crime pay less, a common goal of societies around the world.

The remainder of this paper is organized as follows. The following section highlights the relevance of analyzing money laundering by discussing the volume of money laundering and its harms. Section 2 sets out the conceptual framework for the analysis. Sections 3 and 4 discuss the quality and price of laundering. Section 5 describes what we know about competition and cooperation in the market. Sections 6 and 7 focus on the regulation of money laundering and market developments. Section 8 concludes.

## 1. The Volume of Money Laundering and Its Harms

Since the goal of money laundering is to go unnoticed or to be seen as some other form of legitimate transaction, it is, by nature, difficult to provide credible estimates of how big the problem is. Moreover, the purchase of laundering services is generally a consensual crime: Both parties voluntarily enter into the transaction. Consensual crimes lack a direct victim to report the crime, which means the crime is revealed only when it is actively investigated.

There are a number of estimates of the volume of money laundering. These range in credibility; some lack empirical backup altogether [e.g., the widely quoted International Monetary Fund estimate of 2 to 5% of global GDP (3)], while other estimates are generated with specific economic models (4–7). It is hard to test the accuracy of these estimates. Triangulation of the different estimation methods has not yet been done. With these caveats in mind, most money laundering estimates stay in the range of 2 to 5% of global GDP.

Improvements in the estimates of the volume of money laundering are feasible with a better understanding of the proceeds from crime around the world. Unfortunately, no country has strong measures of crime proceeds, and most countries lack any estimates at all (8).

The volume of money laundered is driven by the number of crimes, the profitability of those crimes, and the extent to which the offender needs to launder the criminal gains. Criminals who are unable to even make minimum wage usually cannot afford luxury items and therefore have much less need for laundering. Spending criminal money in supermarkets and other stores for basic needs will not raise any suspicion and therefore does not require any laundering.^†^ See (9) for an analysis of the laundering needs for different actors in an illegal drug market.

When money laundering is executed successfully, it should leave no trace at all. This can cast doubt on the rationality of criminalizing laundering in the first place. A useful principle for criminalization is the harm principle, as described by John Stuart Mill (10): Since we want as much freedom as possible in our society, victimless crimes should not be criminalized. Money laundering often does not have a direct victim; a business acting as a front for laundering can even be positive for society. The reason to criminalize money laundering is not the direct harm it causes to others but rather that money laundering aids and abets the commission of other crimes (such as drug trafficking, fraud, and cybercrime). As a matter of justice, we would like to deny people committing those other crimes the ability to enjoy the criminal proceeds.

There are thick reports on why money laundering should be illegal, mentioning many potential negative effects such as unfair competition, the undermining of political institutions, price inflation, and the facilitation of other crimes (3, 11–14). However, many of the reports provide no source at all or refer to each other; the empirical substantiation about these effects is lacking (15) (This conclusion about the literature in 2013 still holds today; there have not been sufficiently important contributions to this literature since). Indeed, one can also note the potentially positive economic effects of laundering. Fake profits can be taxed. During a financial crisis, criminals can provide the liquidity banks need to prevent bankruptcy (16). When crimes are committed in country A and laundered in country B, all kinds of intermediaries in country B can profit from facilitating the laundering. It can also provide an inflow of capital for country B. Even though criminal money is seen as morally bad and legitimately earned money as good, in terms of the positive economic effects of an inflow of capital for a country (including the availability of credit, the multiplier effect for economic growth, and the fiscal income for the government), the moral difference does not matter. The main motivation for fighting money laundering should be to control its predicate crimes (17). Predicate crimes are all crimes relevant to anti–money laundering programs. Countries can specify the predicate crimes with a list of crimes, but countries can also adopt an all-crimes approach (18).

To help the “war on drugs,” the United States introduced the Money Laundering Control Act in 1986. This started a “war on drugs money” (19). Criminalizing money laundering gives law enforcement a second chance to catch the criminal, or at least freeze or confiscate the proceeds (15, 20, 21). Even if this fails, money laundering at least makes it costly (in terms of money and effort) to enjoy criminal proceeds. Then, crime pays less, achieving a common goal of societies around the world.

## 2. A Conceptual Framework for the Money Laundering Market

Money laundering is an activity, often a chain of activities, to make the criminal proceeds appear legal. This paper will depart from the legal definition of money laundering and focus on a more criminological, economic, and practical definition, in which money laundering is one or more activities to give criminal proceeds an apparent legal origin.^‡^

The laundering can be done by the criminal themself (called self-laundering) or by a provider of laundering services (called a professional launderer or facilitator). This paper argues that self-laundering is not part of the money laundering market. Self-laundering can be seen as the illegal counterpart of what Gary Becker and Jacob Mincer call household production: Instead of buying a cake from the bakery, one can also buy eggs, sugar, and flour and produce the cake at home with some time and effort (22). The time and effort spent to bake the cake is not counted in standard national accounting statistics; it is not considered to be part of the economy (the market) in that sense. Most laundering cases tend to be about self-laundering (23). However, professional money laundering cases can be harder to detect and might therefore show up less frequently in criminal cases, not reflecting their true proportion of laundering events (see also ref. 24). The advantage of self-laundering is that the criminal does not lose control of the criminal proceeds and therefore does not need to trust another criminal—namely, the launderer. However, self-laundering requires a certain expertise and cannot use licensed professionals (unless the criminal can get the license for themself). Self-laundering, therefore, tends to be less sophisticated and more appropriate when criminal proceeds are smaller. Self-laundering also has the disadvantage that it can be harder to obfuscate the link between the criminal and the proceeds when all the handling of the money is done by the criminal. Since the goal of laundering is to make money appear legal, eventually, the use of legal goods or services is needed, such as using a real estate agent, bank, or notary for a real estate purchase. One cannot make dirty money clean only by using people and organizations who are purely criminal. Somewhere along the line, legal (financial) institutions or transactions have to be involved. Self-laundering does not have a clear distinction between supply and demand, and there is no clear price mechanism. This paper therefore focuses on a laundering market in which a party other than the criminal (a money laundering professional or facilitator) provides laundering as a service. This does not mean that self-laundering is irrelevant for this paper. Self-laundering can be an alternative for criminals when laundering services are not available or are unattractive, and the price professionals can charge for laundering services is influenced by the riskiness of self-laundering.

Many authors (e.g., refs. 25, 26) introduce money laundering with a standard three-stage model of placement, layering, and integration. In the placement stage, the launderer introduces illegal cash profits into the financial system. In the layering stage, the launderer engages in a series of conversions or movements of the funds to distance them from their source. Then, in the integration stage, the funds reenter the legitimate economy (27). This original three-stage or three-phase model is a good example of a complete money laundering cycle (especially in the 1980s and 1990s), but it is a bad description of the general phenomenon. Indeed, some laundering processes have these three stages. However, looking for these stages distracts and confuses because these stages are too often not present in laundering cases nowadays (see e.g., refs. 28–31). Money laundering has evolved since the 1980s from being mostly drug proceeds in cash funneled through bank accounts to more complex and more diverse international money laundering methods with proceeds that are never in cash in the first place (such as investment fraud) (see e.g., refs. 32, 33).

Van Duyne (30) and Matanky-Becker and Cockbain (29) did the most relevant studies on the applicability of the three-phase model. Van Duyne (30) studied 49 observations of money laundering in criminal files in the Netherlands and concludes that the three-phase model, or variations of it, are not helpful to understand laundering behavior. Of the 49 observations, only 12 show signs of placement, only 2 look like integration, and layering is not observed at all. Although placement is observed incidentally, van Duyne also notes that the “placement phase could better be termed ‘*displacement*’, as the concern of the business crime-entrepreneurs was to get the money out of the financial system.” (30) Matanky-Becker and Cockbain (29) studied 31 money laundering investigations in the United Kingdom with 52 suspects. They conclude that their results “undermine the utility and validity of the ubiquitous three-stage model.” (29) Of the 31 cases, only one case had evidence of *all* three stages supposedly central to money laundering, while 21 cases had no evidence of *any* of the three stages.

An outsider trying to understand whether certain behavior is money laundering using the classic three-phase model would be looking for phases that too often do not happen (anymore) and observe money laundering behavior that cannot be classified in any of the three phases. To understand money laundering better today, the three-phase model should be phased out.

A complete laundering cycle generally takes multiple different steps. Confusingly, each of these steps is itself called money laundering, not only in the law but also in the academic literature. Consider a specific laundering example from the Eureka investigation (see ref. 34): Crime money is made in Italy by selling drugs. The cash proceeds are moved to Germany. In Germany, the money is put into a bank account disguised as the cash profits from a car wash. This money can then eventually be spent by the Italian criminals. If the authorities detect the cash being moved to Germany, then they can make a money laundering charge. But if the international movement of the cash succeeds without detection, the money is not laundered yet. Only the next step—depositing the money as car wash proceeds—makes the money appear legal. So, although it is legally correct to give a step such as international cash movement, the label of money laundering, it is analytically confusing that such a step does not actually launder the money.

This paper suggests making a clear separation among concepts that have so far all been called money laundering. The whole laundering process, from criminal proceeds to a situation in which these proceeds cannot be traced back to the crime, will be called a complete money laundering *cycle*, which can consist of one or more money laundering *steps*. The launderer can charge a fee for a complete money laundering cycle or one of the money laundering steps (such as smuggling cash across a border). The overall price for money laundering is then the price for the complete laundering cycle paid at once or the sum of the prices for all the necessary laundering steps. Unfortunately, the limited literature on money laundering prices (e.g., ref. 35) has not made this distinction; the price of one step is often incorrectly called the price of laundering. This makes it hard to interpret current (total) market prices for laundering, even with the empirical evidence available.

There are many ways to launder money (34, 36). One can think of legal ways to become rich and think of laundering methods that can mimic them. There are laundering methods faking any or all of employment, running a business, trade, lottery winnings, gambling, stock market trades, investments, and loans. The customer buying the laundering service does not always know the steps of the laundering process. The money (either cash or electronic) might be sent multiple times around the world, but it could also never leave the town in which the crime money was made.

Cryptocurrencies were initially seen as a game-changer for money laundering. However, in essence, the use of cryptocurrencies is not revolutionary. Launderers have always been interested in changing the form in which the criminal proceeds are held: from cash to a bank account, from luxury items to real estate, and from one currency to another. Cryptocurrencies are just another way to hold or transact criminal proceeds. Cryptocurrencies can have the advantage of being more secretive, because who owns the wallet that holds the cryptocurrencies can be hidden (especially with cold wallets that are offline on physical devices). On the other hand, transactions with cryptocurrencies tend to be fully public on the blockchain^§^, making them more transparent than most other forms of financial transactions. The international anti–money laundering efforts regarding cryptocurrencies follow the logic of more traditional anti–money laundering policies: Platforms that exchange money for cryptocurrencies (or the other way around) have similar duties as banks and regular currency exchangers (37).

The variety of laundering methods makes the analysis of the money laundering market complex. Product differentiation is important in the market, especially because different predicate crimes generate different types of proceeds and laundering needs (see e.g., refs. 28, 34, 38–40). Hetzel (39) shows that the predicate crime is the strongest predictor of laundering behavior, because it determines what form of illicit proceeds are generated (see also refs. 34, 38, 40). Most drug crimes generate cash and might therefore need an extra step to get the money into the banking system, while the proceeds from investment fraud are generally already in the banking system. Investment fraud poses a different challenge: The victim knows to which account the money was transferred. A solution might be to do the opposite: Take the money out of the banking system and move the proceeds in cash to break the money trail. For example, Matanky-Becker (28) lists two cases in which electronic criminal proceeds were converted to cash, and Kruisbergen et al. (41) show that cybercriminals with proceeds that are initially electronic exchange their proceeds for cash.^¶^

The laundering market consists of a customer (the criminal who made money from a predicate crime), a launderer (the one providing the laundering service, the supplier in this market [These were originally called professional money launderers by the Financial Action Task Force (42)], and all kinds of possible intermediaries.

Customers in the money laundering market can be individuals but are often criminal organizations. Some launderers are professionals who have a legitimate (relevant) job as their main activity, such as notaries, car dealers, or lawyers (43). The complete laundering cycle can be arranged by one person acting as a “banker for the underworld” (44). At the other end of the spectrum, money laundering can involve multiple steps (like a supply chain), each done by many people, such as tellers who convert proceeds into 500 euro notes, which are then smuggled across the border by another set of people (as described in ref. 45). Riccardi and Reuter (34) describe how laundering choices can change depending on the offense generating the criminal proceeds. Nazzari and Favarin (40) suggest that professional launderers are specialized in terms of predicate crimes. Specialization seems necessary for the suppliers of more complex laundering services because the required skills and contacts are hard to use for other forms of laundering.

Law enforcement authorities try to detect and prevent money laundering, primarily as a method of reducing the number of predicate crimes. Private sector entities (such as banks, notaries, and real estate agents) are required to facilitate the detection of money laundering by, among other duties, reporting to a government agency called the Financial Intelligence Unit. This is a public authority that countries around the world, under the rules of a powerful international body known as the Financial Action Task Force, have set up to process the reports from the private sector about (potentially) suspicious transactions. These same private “gatekeepers of the financial system” that have to support the detection of money laundering can also facilitate laundering, accidentally or strategically [For example, each of the ten largest European banks has been fined more than $100 million for money laundering violations (8)].

## 3. The Quality of a Money Laundering Service

The criminal might not know the quality of the laundering service beforehand and might not be interested in it anyway. People regularly buy products and services they do not understand; the most relevant question is whether it works. The unavailability of legal recourse in an illegal market is crucial here (46). What if the quality of the money laundering service turns out to be low (high chance of detection, slow, money lost)? Money laundering can be lengthy and have unexpected costs. A customer in a legal market can return defective products for a refund, and if the seller refuses to refund, the consumer can sue in small claims courts or at least post a negative review online. When the transaction is illegal, it is harder for an injured party to obtain compensation, so the consequences are more severe.

The laundering market has two-sided asymmetric information. Both the criminal and the launderer know something the other does not know. Asymmetric information can have a major impact on a market. The standard example is the “market for lemons” of Akerlof (47), who formally introduced asymmetric information to economics: When only the sellers know the actual quality of cars and use this to price each car, then only bad cars seem to be a good deal for buyers who do not know the car’s quality. The result is a market in which only bad cars are being traded—hence, a “market for lemons.”

Criminals can ask the launderer about the service, but the information provided cannot be trusted. Criminals can get information on the quality either through their own experience or through the experience of other criminals. There might be reviews of launderers on anonymous websites or the dark web, but they cannot be trusted automatically. The struggle to become informed about the quality of a launderer is therefore similar to the situation for many legitimate products before the internet: When a new restaurant opened pre-1980s, you could go to the restaurant yourself or ask a friend who had gone; otherwise, it was hard to know the quality of the food (This is assuming that the restaurant has not been visited yet by a food critic.).

The asymmetric information in the laundering market is two-sided: The launderer has incomplete information about how “hot” the money is. If the criminal is later discovered by law enforcement, the launderer is put at risk as well. This makes the laundering market different from Akerlof’s market for lemons. Money launderers have an incentive not to sell a lemon (which is a laundering service with a higher chance of detection in this case), because this can have negative consequences for themselves as well.

Because the quality of the service is hard to know beforehand and trust can be an issue, criminals might try to keep control of the process while still involving necessary third parties. For instance, a Dutch criminal might pressure a notary to process a real estate transaction with a credible threat, such as hurting the notary’s professional reputation, property, or family members.^#^ Berry, Salinas, and Gundur (25) describe a case in which a drug trafficking organization would threaten legitimate business owners, not demanding payments but demanding that the businesses launder their dirty money (see refs. 43, 48 give other examples of criminals having control over the money launderer).

## 4. The Price of Money Laundering

Money laundering regulation is costly. Cost estimates of antimoney laundering systems are generally made by the industry itself, do not originate from independent academic research, and are not consistent in their results and estimating methods. However, the estimates suggest that combating money laundering is a multi-billion-dollar industry (see e.g., refs. 49, 50). This raises the question of whether this fight is effective and efficient (51).

The price of money laundering should, in a competitive market with complete information, reflect the risk involved when laundering money. The price of laundering can then be a good indicator of the effectiveness of anti–money laundering policies (21). Unfortunately, research on the price of money laundering is in its infancy. The best analysis is by Nazzari and Favarin (40), drawing from 90 US criminal cases from 2007 to 2024 [See ref. 52 for a descriptive analysis focused on prizes for money laundering using false invoicing.]. They find a median commission fee of 9% of the amount to be laundered, with notable price variations across different types of predicate offenses. Cybercrime emerges as the most expensive, with a median fee of 25% (*n* = 9), followed by fraud and drug trafficking, each at 7% (*n* = 20 and 31, respectively). A problem with the analysis is that these laundering cases cannot be compared directly, because we know little about the quality of the service, and especially how much of the laundering process it involves. Laundering money from drugs might require four money laundering steps, each with a 7% fee, while the laundering service for cybercrime might directly represent the complete laundering cycle. In this case, laundering money from drugs would be ultimately more expensive, even if the individual fee in the court data appears lower.

It is hard to draw strong conclusions about the price of laundering with the current empirical evidence (see also ref. 43). Recording the prices mentioned in court cases is a good first step, but, eventually, more information is needed about the quality and completeness of the service. A distinction between different money laundering steps and a complete laundering cycle is essential. We can compare the prices of two money smugglers across a border, but comparing the price of a money smuggler with the price of a lawyer who offers a complete laundering cycle with a whole chain of convoluted activities is comparing apples and oranges.

Another distinction important for money laundering prices is whether the price is defined as the fee for the launderer or the cost for the criminal (see ref. 1). This is especially relevant for laundering methods involving large tax payments and other significant transaction costs. For instance, a launderer might set up a cash-intensive business to launder money, such as a bar or, perhaps even more fitting, a laundromat. Criminal proceeds can be added to the cash register of such a business to make them appear as legitimate revenue. The launderer might ask for a one-time fee for setting up the business and the bank account. We could call this the price for the money laundering service. However, the laundering costs for the criminal are much higher than just this one-time fee. The criminal’s costs include business operations and tax—for instance, if the business is in the United States, there is a 21% federal corporate income tax on the “profits.” These money laundering costs are then expected to be much higher than the initial fee paid to the launderer.

Some costs are unrelated to the laundered amount, such as the fixed costs for setting up a cash-intensive business. Other costs are related to the laundered amount, such as the expected asset recovery, which tends to go up when the amount laundered goes up. Most prices for money laundering seem to be a percentage of the laundered amount instead of a prescribed dollar value (40). This would indicate that the price of laundering is mainly driven by costs related to the laundered amount instead of fixed costs. This is remarkable, since we would expect that the risk of detection is mainly driven by features other than the amount laundered, especially after a certain monetary level. Choosing a money laundering price as a percentage of the money laundered might therefore be mostly “convenient pricing” rather than following any rational reflection of estimated risk. Although there is sufficient literature assuming the rationality of the launderer in calculating risk and reward (e.g., refs. 53–56), most empirical literature documents relatively unsophisticated and basic laundering behavior (e.g., refs. 25, 34, 45).

Criminals are willing to give up a significant share of their proceeds if the money can then be spent freely. Without money laundering, criminal proceeds after a certain amount are almost worthless, because spending this money on legal goods or services raises too much suspicion.^||^ We should therefore expect an inelastic demand for money laundering; criminals would want to buy the service even when the price increases considerably. This would mean that offering laundering services can be very profitable when criminals do not have alternatives. Indeed, we see inefficient and costly laundering choices (see e.g., ref. 45). A limitation on the profitability of laundering is that criminals can always try to launder the proceeds themselves as a form of competition. However, this can be much riskier without the required skills and contacts, and the link with the predicate crime might be uncovered more easily. If not only the laundering is detected but also the link with the predicate crime, then this can be more costly for the criminal. The criminal can then be convicted of money laundering and the predicate crime, potentially increasing the expected punishment considerably (57).

When a criminal can choose between different launderers, the price might be only of secondary concern. Trust is particularly relevant for laundering because one gives away the illegal proceeds. In that sense, laundering is not a transaction with direct exchange; there is a delay, which poses risks for the customer. Therefore, the customer might pick the launderer based on high trust, or has to make an effort to “enforce the contract” (see e.g., refs. 43, 48 for examples).

Money laundering can involve facilitators with a legitimate job and reputation to uphold, such as notaries, lawyers, and real estate agents (see ref. 43 for a broader analysis). The relationship between customer and supplier can then take the shape of a subscription model: The supplier can have a hard time getting out of the relationship because the customer now has leverage with the knowledge about their committed crimes (see ref. 48 for an example). The customer can inform the police about the money launderer with a legitimate job.^**^ This can give the customer an edge in future transactions. We can compare the situation with the business model of selling a cheap printer that can then be refilled only with expensive ink. The special feature of money laundering is that this situation is reversed. It is not the supplier but the customer who acquires the leverage. Therefore, the opposite can happen with the price. The customer can pay extra for the first laundering service and then have leverage to get the service cheaper or even for free. Benson (43) presents multiple cases in which the launderer does not appear to get paid for the services.

This subscription-like client–supplier relationship and necessary trust mean that we can expect that laundering relationships are longer-term. Remarkably, this goes against more general predictions about illegal markets, in which client relationships are expected to be more ephemeral than in legal markets (58).

A characteristic of illegal markets is that information flows are often poor. This affects demand and supply. Advertising and comparison shopping are risky and therefore expensive. Tomorrow’s customers have a hard time observing today’s transactions and prices. Although there is not sufficient empirical evidence in the literature, we expect the dispersion in prices to be greater than in legal markets (as also observed in other illegal markets, see ref. 59), even for equivalent services, and there to be slower convergence of prices to a new equilibrium. That also lets a local monopolist charge higher prices.

## 5. Competition and Cooperation in the Money Laundering Market

The supply of laundering services generally adapts to the type and extent of laundering services needed.^††^ Changes in the laundering market are therefore generally the consequence of changes in the underlying demand. The demand for laundering is driven by the profitability of crimes (when crimes are more profitable, there is more demand for laundering, ceteris paribus), what constitutes a crime (legalizing certain drugs could significantly reduce the demand for laundering), and how hard and risky self-laundering is (in general, the harder it is to self-launder, the more demand for laundering, ceteris paribus).

A criminal can try to do some window shopping. For instance, real estate agents are generally not allowed to share details of suspicious customers with each other. So a criminal can visit real estate agent A and seek the purchase of real estate with criminal proceeds and, when rejected, try again with real estate agent B and so on until one agrees to provide the service [for instance, because a professional is in a desperate financial position, as reported in (43)]. However, criminals cannot go around freely asking for laundering, as this can expose them to the authorities (real estate agents cannot share details with each other, but they can report to the authorities). This inability to comparison shop and a limited information flow can lead to seemingly local monopolies. Even though there might be competitors supplying laundering services, as long as the criminal is unaware of this competition because of the high search costs, the launderer can enjoy a monopoly position and therefore drive up the price because of the inelastic demand. Local monopolies can also exist because launderers tend to specialize in specific predicate crimes: Some launder only proceeds from cybercrime, while others launder only drug proceeds (see ref. 35).

We generally do not see a competitive market with different suppliers offering different prices and quality. However, for some steps in the laundering process, this can be the case. The most competitive laundering markets can be seen when underground bankers and cryptocurrency launderers offer their services.

Traditionally, underground bankers help migrant families send (cash) money back to their home countries, where formal banking systems are underdeveloped. Underground banking is illegal because offering banking services requires a license, but these services are used so widely as an informal banking system that information about these services spreads more easily than about other forms of laundering (60). Although it can be helpful for a criminal to quickly move cash from one country to another, this does not really “clean” the money and can therefore not be seen as a complete laundering cycle, merely one of the needed laundering steps.

Several websites offer the laundering of cryptocurrencies. Since the blockchain registers the path of the cryptocurrency from the moment it was mined to the current moment, it can be valuable for criminals to “break” that path. Simply put, the idea of a cryptocurrency laundering website is that the user can send their (illegal) cryptocurrency to the website and receive other cryptocurrencies back, minus a fee (61). This seems like a competitive market with professional services, though it is mixed in with complete scams that steal cryptocurrency (62). To what extent such a service really “cleans” the criminal proceeds can be debated (63). Yes, the path to the origin of the cryptocurrency might be broken, but that does not provide its owner with a legitimate story on how these cryptocurrencies were legally earned.

Though the markets of underground banking and cryptocurrency launderers show signs of being competitive, with multiple suppliers, different services, and competitive pricing, these are only for individual laundering steps that do not provide a legitimate source for the crime money. There are no signs of competitiveness in the market for a complete money laundering cycle; therefore, it seems safe to assume that these are generally local monopolies.

Money laundering with complex financial instruments can only be done by specialized professionals who tend to already earn a good wage with their legitimate activities. This creates a market with a substantial barrier to entry. On the other hand, an activity such as smuggling cash across a border (relatively low-skilled labor) has a much lower barrier to entry. We can expect a more competitive market for such laundering steps.

It is unclear to what extent launderers are aware of their competition. Some launderers talk about the prices of their competitors to explain their own price (40). This does not mean that they are actually aware of their competitors and their prices; this can also be an easy way to convince the criminal to purchase the laundering service. Limited contacts can create monopoly opportunities in the market and can lower the risk of being ratted out by a competitor. Kramer et al. (64) investigate the contacts of 198 financial facilitators involved in money laundering in the Netherlands in the period 2016–2020. This study shows that basically only two types of launderers are in contact with their peers: underground bankers and real estate agents (64). Other types of launderers (such as financial service professionals and virtual asset service providers) seem to limit their contacts with peers.

## 6. The Enforcement and Regulation of Money Laundering

There is international pressure to fight money laundering, coming primarily from (the members of) the Financial Action Task Force, an intergovernmental body setting standards for anti–money laundering policies. National incentives to fight money laundering differ. For some countries, money laundering regulation can help to fight organized crime in the country; for other countries, anti–money laundering policies are bad for their (banking) business and for attracting foreign capital. Countries mutually evaluate whether each other’s anti–money laundering systems comply with international standards, but these checks struggle to go much beyond compliance on paper (65, 66).

Money laundering can be done everywhere and in many ways. However, some locations have more facilitating tools for laundering, such as corrupt officers, complex financial instruments, offshore companies, and weak beneficial ownership rules. Differences between countries in money laundering enforcement (in practice) can play a major role when launderers decide which countries to involve in their laundering process. For instance, a money launderer explained in a TV interview in 2006 that he regularly drove with a trunk full of cash from the Netherlands to Hungary, because laundering controls in Hungary were deemed less strict than in the Netherlands.^‡‡^

Corruption can be helpful, but it can also be a deterrent to money laundering.^§§^ On the one hand, corruption can help a launderer avoid prosecution if caught, as well as ask the customs guard to look the other way to avoid apprehension in the first place. However, corruption can also be costly, especially because some laundering methods require many transactions, and corrupt regimes can be untrustworthy transactional partners. When legal structures in stable democracies exist that seem willing to facilitate money laundering (see e.g., ref. 67), engaging in corruption might not be necessary. Research so far seems to indicate that less corrupt countries are more attractive for launderers (4, 68). This can be because of lower costs, because less corrupt countries provide more legitimacy to the source of the funds, or because there is a lower expected chance that an agent steals the funds. Risk systems to detect money laundering tend to work with blacklists and greylists, but these lists seem to stem more from political relations, stereotypes, and other crime risks (e.g., corruption) than from actual money laundering risks (see ref. 69).

There are incentive issues with fighting money laundering in an international context. There are three forms of money laundering flows in a country: domestic predicate crime money that is laundered, crime money flowing in from abroad to be invested in the country, and money just flowing through (coming from abroad and flowing through the country on its way to the final destination) (4). These three forms of laundering have very different effects on the country. Domestic predicate crimes harm the safety and long-term growth of the country, while money flowing through might have only a small positive effect by benefiting some related professionals (e.g., bankers, notaries, and lawyers). Countries with relatively few domestic predicate crimes might not be so internally motivated to fight money laundering, even when significant amounts of crime money are flowing through. Fighting such flows will mostly benefit other countries. This makes it understandable that substantial international pressure is needed to convince countries to have a robust anti–money laundering framework in place.

## 7. Market Developments and Dynamics

The money laundering market is generally opaque; prices and the quality of services are not openly discussed. This suggests an impeded flow of information (as 50 notes as a general characteristic of illegal markets). Parties in a volatile, sometimes violent, illegal market will not always act in a stable and rational way. This leads to the expectation that the money laundering market is generally out of equilibrium.

Criminals and law enforcement agencies are in a cat-and-mouse game. Criminals come up with a new laundering method every now and then (e.g., laundering with cryptocurrencies). Regulators and law enforcement agencies detect the new laundering method and try to thwart it (e.g., with regulations targeting virtual asset service providers or setting up investigation teams focused on cryptocurrencies). If these efforts are sufficiently successful, criminals might want to adapt. The forms of money laundering are therefore expected to change over time, particularly as new modes of financial transaction are constantly being developed. If we do not see a change over time, this can indicate that criminals are not sufficiently sophisticated to innovate or that law enforcement agencies are unable to sufficiently mitigate existing laundering methods. Innovation is not necessary when old methods still work. We have seen many innovations and new methods of laundering. However, it is just as interesting that even with all the innovations, we keep seeing old and standard laundering methods still being used [e.g., ^¶¶^ the loan-back method and funneling money through cash-intensive businesses. For example, the Dutch Financial Intelligence Unit reported in 2024 on a case with a loan-back method (see https://www.fiu-nederland.nl/knowledge_base/carriereswitch-van-crimineel-naar-vastgoedondernemer/, in Dutch).] This can imply that criminals see no need to innovate (70). Alternatively, we keep seeing the old methods because they are easier to discover, either because they are used by dumber criminals or because they are more often recognized by law enforcement agencies that are still focused on old laundering methods.

## 8. Concluding Remarks

Money laundering is understudied, given its importance. The sheer quantity of money laundered, the costs associated with controlling it, and the crucial role of anti–money laundering policies in ensuring societal security warrant a more thorough analysis than is currently present in the academic literature.

However, only a small number of researchers focus on the topic. Most important questions do not have clear, well-supported answers. It is, for instance, not clear whether the strengthening of the anti–money laundering framework over the years affected the supply of laundering services, increased the price of laundering, or made societies safer (17, 51). We are only starting to understand some elements of the laundering market. Properly understanding the market can inform policy and enforcement decisions and contribute to a more effective and efficient fight against crime (see ref. 17 for an analysis of the effectiveness of the current global money laundering control system).^##^

Not all forms of laundering, and not all laundering steps, are equally suitable or cost-effective targets for fighting crime. It would be useful to estimate how cost-effective it is to disrupt different steps in the laundering process and use that information to determine which steps are worth targeting. We have to recognize that the optimal focus might change once criminals react and adapt to such policy changes.

The volume of money laundering that occurs can be reduced by making predicate crimes less profitable (clearly not easy to do) or making fewer behaviors a crime (legalizing the supply of certain drugs could significantly reduce the demand for laundering and therefore the volume laundered, for example). Successfully fighting money laundering might not directly affect the volume of money laundering, but it can increase the price of laundering and therefore the cost for criminals. Competently fighting money laundering will at least catch some criminals who were not caught for the predicate crime and make crime pay less for those who need to purchase more expensive laundering services. When criminals launder their criminal proceeds by reporting them as legal income, even when successful, criminals at least make an extra tax payment on those reported profits (25, 71).

One of the policy choices is whether to fight predicate crimes directly or indirectly with money laundering controls. An analysis of which method is most cost-effective could inform this decision. The cost-effectiveness studies so far (e.g., refs. 72, 73) have not been able to answer this policy question.

Further research on the money laundering market has to recognize that, in criminal cases, many different behaviors are all labeled as laundering. For a better understanding of the laundering market, different forms and steps of money laundering need to be separated to avoid comparing apples and oranges.

This paper relies on an economic analysis of a market and with that implicitly assumes (some level of) rationality by the criminal and money launderer. Though there are plenty of other studies that assume the rationality of criminals (e.g., refs. 74–76) and launderers (e.g., refs. 54–57, 77), one can wonder how rational and organized criminals and money launderers are in practice (2).

## Data Availability

There are no data underlying this work.

## Footnotes

^*^Hiding the illegal source can help to avoid detection, for instance, because a private party might be less likely to report to the authorities when there are no signs of an illegal source. However, an apparent legal source is generally needed once authorities start asking questions about the origin of the money. Although hiding the illegal source can help, and a criminal can enjoy their ill-gotten proceeds when authorities do not ask any questions, money is really laundered only when it is given an apparent legal source. This paper focuses on the process of giving illegal money an apparent legal source and, from now on, leaves out the nuance that in some situations hiding the illegal source is sufficient. Money laundering does not concern only money but also all other types of proceeds. For readability, this paper sometimes uses *money* and *proceeds* interchangeably.

^†^This form of money laundering is sometimes called *passive money laundering* (see e.g., ref. 25). The volume of the criminal proceeds that needs to be laundered and the volume that can be spent freely on leisure and subsistence expenditures are context-specific. There is no strong empirical literature determining these ratios. One study (9) calculates these ratios in specific scenarios; others (5, 6, 78) extrapolate such ratios based on simplistic expert elicitation (see ref. 79).

^‡^The legal definition of *money laundering* differs across jurisdictions, but can be very broad, making it hard to define the laundering market well (see ref. 65). In some countries (like the Netherlands, Luxembourg, and Cyprus), following the strict legal definition, having crime proceeds is considered laundering even before anything is actively done with the money (see ref. 65). This means a bank robber walking out of the bank can technically be a launderer, simply because they have the crime money. However, analytically, we should classify this only as a bank robbery. In countries with a somewhat narrower definition (like the United Kingdom and Bulgaria), hiding criminal proceeds is considered money laundering. Then, a bank robber who did nothing more than bring the money home and hide it can be accused of laundering money. Also then, analytically, we should classify this only as a bank robbery.

^§^The blockchain is basically a public and decentralized administration system tracking cryptocurrency transactions. The blockchain is a bunch of characters clustered in blocks chained together. Each new block contains a list of the recent transactions. When crypto is transacted, this transaction is verified and added to the chain. Once a transaction is added to the chain, it cannot be changed anymore. This makes it possible to verify whether someone sending crypto has enough crypto to do so, and to avoid the same crypto being spent twice at the same time.

^¶^Known cases of money laundering are so fragmented that it is currently not possible to give a representative overview of money laundering cases or understand what is typical and what is uncommon (33). The case descriptions in this paper should therefore not be interpreted as such. A case reference generally highlights an empirical observation that alludes to a point or provides a relevant example, without claiming it can be used to draw general conclusions.

^#^Contract enforcement is a general issue with criminal business transactions. When illegal transactions do not occur as agreed, it is not attractive to involve the authorities. When millions of drug proceeds were stolen from Piet Costa (an alias for a famous Dutch criminal) by someone within his organization, he did not go to the police, as other victims of theft would have done. Instead, his organization built a torture chamber to set things “right.” Eventually, the police got hold of this chamber when a picture of it was shared via encrypted communication EncroChat (later decrypted by the police). The planned torture was prevented, and 11 men were convicted, but the news about this torture chamber shocked Dutch society (80).

^||^The criminal proceeds can still be used to buy illegal goods or to fund other illegal activities (like bribing a politician). The assumption is that this is valuable only up to a certain amount. After that, the criminal is mostly interested in legal goods and services.

^**^This can also put the criminal, or their criminal proceeds, at risk. This threat is most credible when the professional money launderer with a legitimate job has more to lose than the criminal.

^††^There are incidental examples of changes on the supply side that affected the market, such as the emergence of Chinese suppliers offering false invoicing services (see ref. 57).

^‡‡^The Dutch TV program Zembla (season 12, episode 22, September 17, 2006) interviewed relevant stakeholders about money laundering, among whom was an anonymized Dutch money launderer. Available online (in Dutch): www.bnnvara.nl/zembla/videos/188419.

^§§^Leaders of corrupt countries can try to systematically loot the resources of their country. These kleptocrats can launder such proceeds in their own country because of their control of local institutions. This is a specific situation that this paper does not focus on. See (81) and (82) for discussions of how kleptocrats can move proceeds outside the country of origin.

^¶¶^The loan-back method is a relatively well-known and classic money laundering method. Though the details can differ, the idea is that the criminal loans their own illicit funds back to themself. To the outside world, it should appear to be a loan by a third party, while the third party is in fact the criminal himself (possibly via an offshore company, a trusted family member, or friend) (83–85).

^##^An effective fight against crime also depends on factors that are not the focus of this paper, such as political will, relative priority to other policy goals, and sufficient capacity for law enforcement agencies.
